# An iterative consensus-building approach to revising a genetics/genomics competency framework for nurse education in the UK

**DOI:** 10.1111/jan.12207

**Published:** 2013-07-23

**Authors:** Maggie Kirk, Emma Tonkin, Heather Skirton

**Affiliations:** Genomics Policy Unit, President-elect, International Society of Nurses in Genetics, Faculty of Life Sciences & Education, University of South WalesGlyntaf Campus, Pontypridd, UK; Genomics Policy Unit, Faculty of Life Sciences & Education, University of South WalesGlyntaf Campus, Pontypridd, UK; Chair, European Board of Medical Genetics, Plymouth UniversityUK

**Keywords:** competence, competency, consensus approach, education, genetics, genomics, nursing

## Abstract

KIRK M., TONKIN E. & SKIRTON H. (2014) An iterative consensus-building approach to revising a genetics/genomics competency framework for nurse education in the UK. Journal of Advanced Nursing 70(2), 405–420. doi: 10.1111/jan.12207

**Aim**To report a review of a genetics education framework using a consensus approach to agree on a contemporary and comprehensive revised framework.

**Background**Advances in genomic health care have been significant since the first genetics education framework for nurses was developed in 2003. These, coupled with developments in policy and international efforts to promote nursing competence in genetics, indicated that review was timely.

**Design**A structured, iterative, primarily qualitative approach, based on a nominal group technique.

**Method**A meeting convened in 2010 involved stakeholders in UK nursing education, practice and management, including patient representatives (*n* = 30). A consensus approach was used to solicit participants' views on the individual/family needs identified from real-life stories of people affected by genetic conditions and the nurses' knowledge, skills and attitudes needed to meet those needs. Five groups considered the stories in iterative rounds, reviewing comments from previous groups. Omissions and deficiencies were identified by mapping resulting themes to the original framework. Anonymous voting captured views. Educators at a second meeting developed learning outcomes for the final framework.

**Findings**Deficiencies in relation to Advocacy, Information management and Ongoing care were identified. All competencies of the original framework were revised, adding an eighth competency to make explicit the need for ongoing care of the individual/family.

**Conclusion**Modifications to the framework reflect individual/family needs and are relevant to the nursing role. The approach promoted engagement in a complex issue and provides a framework to guide nurse education in genetics/genomics; however, nursing leadership is crucial to successful implementation.

**Why is this research needed?**

- The scale and pace of developments in genomic health care have implications for all areas of nursing and timely review of existing genetics education guidelines is good practice.
- Nurses lack competence and confidence in genetics/genomics and the existence of a competency framework is a critical factor in the integration of genetics/genomics into nurse education and practice.
- The original UK genetics education framework did not take a holistic view of genomic health care and this required attention, to reflect also the integration of genomics into mainstream health care.

**What are the key findings?**

- The original competency statements were found to remain relevant to patient/family needs, but the themes of Advocacy, Information management and Ongoing care were inadequately addressed within it.
- A new competency statement was developed to articulate the nursing role in provision of ongoing care and support to patients, carers and families with genetic/genomic healthcare needs.
- The revised framework is focused on individual/family needs and takes a holistic view of the implications of genetics/genomics for individuals and families across the life stages and the care pathway.

**How should the findings be used to influence policy/practice/research/education?**

- Strong leadership is needed in the UK to embed the genetics/genomics framework into nurse education so that nurses at all levels of practice are competent to meet individual/family needs.
- The novel consensus approach may be useful to nurses in other countries for reviewing existing genetics/genomics competency frameworks, or for developing/reviewing guidelines in other areas of clinical practice.

## Introduction

The first genetics education framework for nurses and midwives was developed in the UK, by consensus amongst an expert group (*n* = 40) and subsequent wider consultation with stakeholders (Kirk *et al*. 2003). Endorsed by the UK nursing regulatory body, the Nursing and Midwifery Council (NMC), the framework set out seven competencies representing the minimum standard that should be achieved by nurses and midwives on qualifying (Table S1). The competencies centred around identifying people who might benefit from referral to genetics specialists. Recognizing the pace of genetics/genomics research and the need for nurse education and training to reflect the changing face of health care, the authors recommended that review of the framework take place within 5–10 years. Advances in genomic health care since then have been of a significance not predicted in 2003 (House of Lords Science & Technology Committee 2009), while the NMC has also revised its pre-registration nursing education standards (Nursing & Midwifery Council 2010). It was thus timely to review the framework, providing separate statements for nurses and midwives, to ensure that each remains relevant to individual/family needs and the professional role. A national meeting involving UK nurses in practice and management, educators, policy makers and patient representatives was convened in 2010 to undertake this task. The authors of this paper consider that process and the subsequent revision of the genetics education framework for nurses and:

- Outline the rationale for review in the context of advances in genomic health care and professional policy and practice;
- Present a method for reviewing an existing education framework with an expert group in an interactive, iterative approach, using real-life stories to strengthen the validity of any consensus reached;
- Present the revised framework and the changes deemed important to reflect the genetic/genomic healthcare needs of individuals, patients and families that are relevant to the nursing role.

## Background

### Concept of competence in nursing

Debate and controversy on the concept of competence in nursing practice has existed for well over two decades, with inconsistencies and a lack of clarity over its definition (Cowan *et al*. 2007, Garside & Nhemachena 2013, Smith 2012). Cowan *et al*. (2007) argue that an holistic definition needs to be agreed on, whereas in their analysis, Garside and Nhemachena (2013) emphasize the importance of context in any specific definition. In her concept analysis, Smith (2012) observes that a benefit of the ongoing debate has been that the concept of competence has been comprehensively explored from multiple perspectives and highlights the importance of seeing competence as a journey where levels of competence develop over time.

For the purposes of this paper, we have used the definition of competence stated by the NMC: ‘The combination of skills, knowledge and attitudes, values and technical abilities that underpin safe and effective nursing practice and interventions’ (Nursing & Midwifery Council 2010, p45). We use the term ‘competency’ to refer to the description of the elements required to demonstrate competence.

### Rationale for review

#### Advances in genetics

Genetics is the study of heredity and variation. Genetic health care is associated with single gene and chromosomal conditions traditionally managed in specialist genetics services (Task & Finish Group 2011). Genomics is the study of the structure and function of the genome, including the interaction between genes and between genes and the environment. By 2010, a decade after the publication of the first draft of the human genome, there had been a dramatic increase in knowledge of the potential contribution of genes to disease prevention and treatment, including common conditions, although the consequences for clinical medicine were as yet modest (Varmus 2010). The rapid advances were fuelled by developments in DNA technologies, including a radical reduction in both time and cost of sequencing an entire human genome (Wright *et al*. 2011). Feero *et al*. (2010) outline how, in the practice of genetic medicine, the pace of discovery has yielded useful information to improve clinical management of single gene conditions. The growing understanding of gene–gene and gene–environment (including epigenetic) interactions underpinning the common multifactorial conditions is also leading to advances in genomic medicine. The Human Genomics Strategy Group (HGSG) describes genomic medicine as ‘patient diagnosis and treatment based on information about a person's entire DNA sequence, or genome’ (Human Genomics Strategy Group 2012, p14). It notes the transformative effects of developments in genomic technology on mainstream health care, offering improvements in speed and accuracy of diagnosis, clarifying predictive risk, informing therapeutic options and supporting public health programmes (Human Genomics Strategy Group 2012). Genomic health care takes a broader view, involving ‘the use of genomic information and technologies at any stage of the healthcare continuum to determine disease risk and predisposition, diagnosis and prognosis and the selection and prioritization of therapeutic options (Task & Finish Group 2011, p6). It also takes into account the associated potential ethical, psychological and social (including lifestyle) implications.

Whilst the impact of genomic research in oncology is well-documented, Burton (2011) conducted a detailed consideration of its impact on two other mainstream specialties, cardiology and ophthalmology. However, with the molecular basis for over 2700 disorders now known, Burton (2011) argues the potential for genetics/genomics across most areas of health care. She emphasizes the importance and necessity for clinical specialties to integrate genetics/genomics into practice, working closely with specialized genetics services. Authors of a review of models of health care in relation to genetics services across Europe, North America and Australia reached a similar conclusion (Battista *et al*. 2012). The scientific imperative for the provision of an appropriate genetics/genomics education framework for nurses is strong.

#### Developments in UK nursing policy

Several UK policy initiatives related to nursing have raised the profile of nurses' competence in genetics, highlighting the need to ensure that education guidelines are relevant and adequate. The House of Lords Science and Technology Committee's (2009) review of genomic medicine urged the NMC to ‘set detailed standards across the curriculum on genetics and genomics for nurses, both for pre-registration nursing education and as part of postregistration education and practice’ (section 7·24). However, in its revised requirements for pre-registration nurse training, the NMC makes one limited reference to genetics, stating that all nurses should take genetic, environmental and other factors into account when conducting a comprehensive, systematic nursing assessment (Nursing & Midwifery Council 2010). No indication is given of how any genetic factors identified should inform subsequent planning and care, nor the knowledge and skills needed to underpin this. Whilst the acknowledgement of the relevance of genetics to a nursing assessment is welcomed, it falls short of the recommendation of the House of Lords.

The Task and Finish Group (2011) report to the UK Nursing & Midwifery Professional Advisory Board gave a very clear indication that the profession needs to do more in leading and embracing genomic health care in nursing. In relation to education, it recommended that NMC standards should be expanded, providing more explicit guidance on curricula, informed by new and existing competency frameworks. All 12 recommendations were accepted by the Professional Advisory Board and subsequently endorsed by the Human Genomics Strategy Group (2012).

### International context

The need for nurses to become more engaged with genetics/genomics and the importance of implementation of agreed core competencies are acknowledged internationally. Skirton *et al*. (2010) stressed the need for common minimum standards for health professionals across Europe. Following a thorough consultation, they compiled core standards in genetics for several health professional groups, those for nurses and midwives building on the UK nursing framework (Kirk *et al*. 2003). Nurses in the USA, inspired by the UK approach, established their essential nursing competencies in genetics and genomics in 2006 (Jenkins & Calzone 2007). Vigorous leadership has continued to drive the implementation of the framework (Calzone *et al*. 2011). In their review of the status of genetics/genomics in nursing across 10 countries, Kirk *et al*. (2013) identify a genetics competency framework as one of the critical factors in integrating genetics/genomics into nurse education and practice, with competencies established as part of a country's regulatory requirements acting as a catalyst for further progress.

#### Current levels of nursing confidence and competence in genetics

Smith (2012) notes that a competent nurse should feel confident; however, a confident nurse is not necessarily competent. A few studies have assessed nursing confidence and competence in relation to genetics, but the evidence is limited. Kirk *et al*. (2007) examined nurses' confidence in being able to demonstrate each of the seven competencies in the UK genetics education framework. Confidence was variable; at most, 48% of respondents (*n* = 198) reported that they felt ‘very confident’ in relation to one of the competencies. Frequencies of those who felt ‘not at all confident’ ranged from 13–63% for the seven competencies. At least 22% of respondents applied each competency on a monthly or weekly basis. There was a significant positive correlation between frequency of competency use and perceived confidence.

In their systematic review of nurses' competence in genetics, Skirton *et al*. (2012) retrieved 13 papers reporting data on 11 studies between 2000–2011. Knowledge of genetics concepts and related clinical skills was generally poor across the five countries represented. A recent study on genetics knowledge and clinical comfort among Taiwanese nurses (*n* = 190) found that although most reported ‘some’ to ‘high’ knowledge of genetic terms, the majority indicated ‘none’ or ‘minimal’ knowledge about genetic conditions and limited comfort in relation to clinical tasks (Hsiao *et al*. 2012). An online survey using a convenience sample of practising nurses in the USA (*n* = 239) identified poor levels of knowledge and confidence in their genetics knowledge and skills (Calzone *et al*. 2013). Response rates to individual questions varied: only 19% of respondents (*n* = 24/126) rated understanding of the genetics of common diseases as good or very good, 16% (*n* = 14/90) felt confident in drawing a family tree and just 6% (*n* = 9/158) felt very confident about facilitating referral to genetics services. There is clearly a deficit in nursing knowledge and skills in relation to genetics and competency frameworks can play a part in driving the incorporation of genetics into nurse training (Feero & Green 2011).

### Consensus approaches

Consensus approaches, particularly Delphi, and nominal group techniques have long been used in a variety of health settings to solve problems and reach agreement on challenging issues (Fink *et al*. 1984) and to develop clinical guidelines (Murphy *et al*. 1998). Delphi techniques have been commonly used to develop nursing competencies, for example, for renal nurses in Sweden (Lindberg *et al*. 2012), emergency nurses in Australia (O'Connell & Gardner 2012) and family practice nurses in Canada (Moaveni *et al*. 2011). Gibson and Soanes (2000) used the nominal group technique to develop clinical competencies for paediatric oncology nurses, whilst McCance *et al*. (2012) used it in conjunction with a consensus conference to identify key performance indicators for nursing and midwifery. Other approaches have been used, often combining several consensus techniques (Murphy *et al*. 1998). Glaser (1980) describes a state-of-the-art consensus approach to develop best practice guidelines. Landeta *et al*. (2011) propose a Hybrid Delphi technique seeking to resolve some of the problems associated with Delphi and nominal group techniques, combining both approaches with focus groups. Homer *et al*. (2012) describe a national consensus approach to developing a competency model and education framework for primary maternity services in Australia, using a combination of a Steering Committee, a wider Reference Group and public consultation.

A rigorous approach to reviewing an existing framework was required that would yield results in a relatively swift timescale and not be too time-consuming for participants. Opportunity for face-to-face dialogue between professional groups and lay individuals was important for information exchange and clarification across a diverse group to reach shared understanding.

## The study

### Aims

The aim of this project was to review the existing genetics education framework to ensure that it was relevant to current nursing practice and appropriate to meet the healthcare needs of people affected by genetic conditions or specific genetic risk. The objectives were thus to ascertain:

- if the competencies in the genetics education framework remained relevant for nursing practice and patient care;
- if there were any omissions or deficiencies;
- if they needed to be refocused in the light of scientific and policy advances or changes in service delivery.

### Design

**Figure 1:**
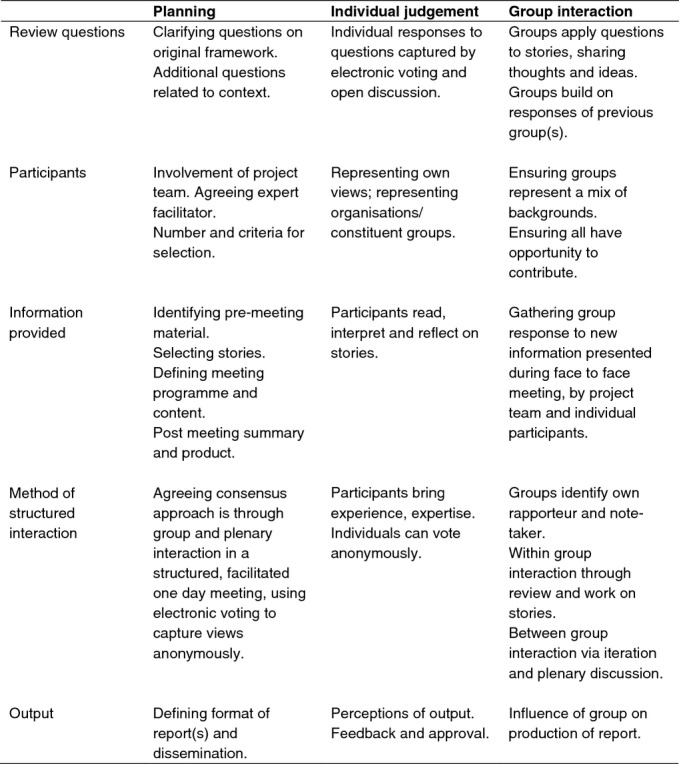
Conceptual framework for the review.

A systematic, interactive, iterative, primarily qualitative approach, based on a nominal group technique involving a face-to-face meeting of invited stakeholders was used, with e-mail follow-up. The underpinning conceptual framework (Figure 1) was informed by the model of Murphy *et al*. (1998) for consensus development methods. Electronic voting technology was used to capture views and assess consensus anonymously. A consensus threshold of 75% was set prior to the meeting.

### Participants

Participants invited to the review meeting had to satisfy criteria agreed by the project team. Patient representatives were selected from a network of storytellers with experience of genetics services, either due to their own health status or because of caring for someone with an inherited condition. Others had to represent one of the professional stakeholder groups identified, with expertise acknowledged by virtue of their professional/public role. Apart from those representing specialist genetics services, expertise or experience in genetics was not a prerequisite as the team wanted to capture a broad perspective. The stakeholder groups (not mutually exclusive) were all four branches of nursing and nurses in practice, management, education and research, the Royal College of Nursing (RCN) and the NMC. All four countries of the UK were represented.

### Selection of stories

An important element of the approach was using real-life stories from individuals affected by genetic conditions or risk to guide and inform the review. Preparing scenarios was an option, but potentially could have introduced bias. Stories were selected from the Telling Stories website (http://www.tellingstories.nhs.uk), a genetics education resource (Kirk *et al*. 2011). Personal accounts are recorded verbatim and mapped to genetics education frameworks and other criteria through a searchable database. Stories have been collected from 2005 to the present.

Stories were selected against agreed criteria, ensuring that collectively they would span all branches of the major nursing specialisms in the UK (adult, child, mental health and learning disability nursing) and life stages (antenatal, infant/child, young person, adult, older person, end of life). Twelve were shortlisted from which six were chosen reflecting as broad a perspective and clinical diversity as possible (Table 1).

**Table 1:** The stories used in the review

Storyteller	Condition	URL
Non	Muscular dystrophy (skeletal muscle disorder)	http://www.tellingstories.nhs.uk/stories.asp?id=76
Diane	Familial adenomatous polyposis (multisystem disorder)	http://www.tellingstories.nhs.uk/stories.asp?id=2
Jonathan	Familial hypercholesterolaemia (high blood levels of low density lipoprotein)	http://www.tellingstories.nhs.uk/stories.asp?id=72
Kiran	Fragile X syndrome (developmental delay and cognitive impairment)	http://www.tellingstories.nhs.uk/stories.asp?id=33
Karen	Tuberous sclerosis (multisystem disorder)	http://www.tellingstories.nhs.uk/stories.asp?id=5
Nancy	Huntington disease (neuro-psychiatric condition)	http://www.tellingstories.nhs.uk/stories.asp?id=69

### Data collection

#### The review meeting

**Figure 2:**
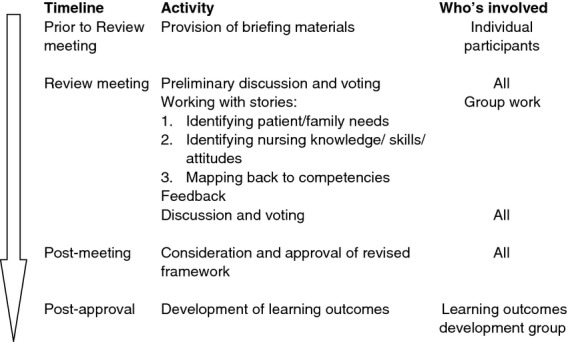
The review process.

The review programme was structured, with clearly identified stages (Figure 2). Briefing material issued to participants beforehand included the six stories and a summary of the existing genetics framework. An expert facilitator from outside the fields of nursing and genetics managed the discussion. This was an important role, given the mix of people present and the potential for strong personalities to dominate. Group discussions were facilitated by project team members. Preliminary discussion and voting focused on awareness of the framework prior to the review and views on nurses’ levels of competence in genetics. Participants were then allocated to one of five groups, ensuring that each had a mix of stakeholders. Each group was provided with a worksheet containing a patient/carer story, with two questions to consider:

- What are the patient/client needs? (including family members and carers)
- What does the nurse need to know, think and do to meet those needs?

**Figure 3:**
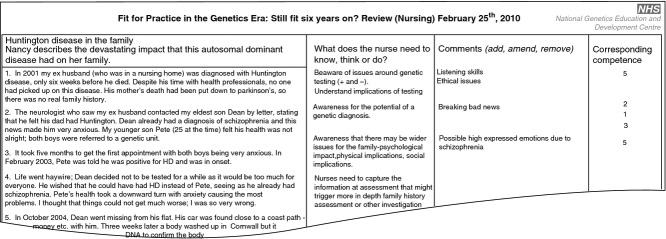
Sample of a story worksheet.

**Figure 4:**
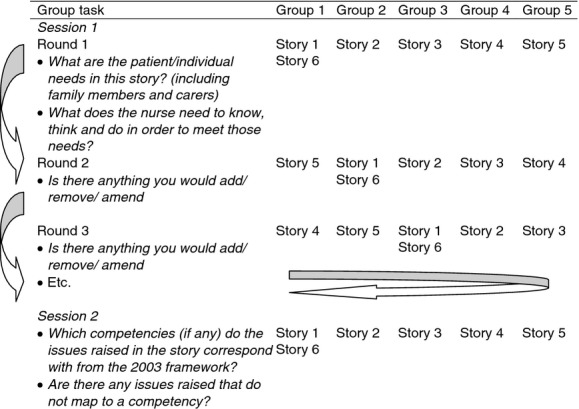
The iterative review process.

After discussing question 1, responses to question 2 were captured on a worksheet (Figure 3), taking a participative thematic analytical approach. Stories with accompanying worksheets were rotated between the groups for review and additional comments. Each story was reviewed by at least four groups (Figure 4).

Groups then reviewed their first allocated story, comparing all responses against the original framework's seven competency statements, identifying elements requiring updating, deficiencies, omissions and any other issues. During plenary discussion, rapporteurs for each group highlighted key issues. Emerging themes were recorded on a flip chart and overlapping or similar/key categories identified for further discussion and voting. Participants voted on whether each category warranted a new competency statement; could be included more explicitly in existing statements and, if so, which one; or should be excluded. Finally participants' views were sought on the draft-revised NMC pre-registration nursing requirements in relation to genetics (Nursing & Midwifery Council 2010).

#### Completing the framework

Following the meeting, the project team amended the original competencies to reflect the discussion. Revision summaries and the updated framework were e-mailed to meeting participants within 7 weeks for further comment and approval.

#### Developing Learning Outcomes

Experienced nurse educators attended a second meeting (*n* = 6), three of whom participated in the review meeting. Working with the new statements, the group developed learning outcomes and practice indicators for each of the eight competencies, building on those in the original framework and on the work of Skirton *et al*. (2006). The Quality Assurance Agency for Higher Education (The Quality Assurance Agency for Higher Education 2008) Framework was used to guide development.

### Data analysis

Issues emerging from the stories were identified through a participative thematic analytic approach. Themes highlighted by rapporteurs were collapsed into major categories through participant validation. Following the meeting, two team members independently reviewed the field notes and flip chart data alongside the worksheets to ensure congruence. Descriptive statistics were used for the voting data.

### Ethical considerations

Detailed invitations were sent at least 4 weeks prior to the review meeting. Further details, including the programme and stories, were distributed at least 1 week beforehand. Participation was voluntary with consent deemed to have been given by attending. The UK Central Office for Research Ethics Committees notified the National Health Service (NHS) National Genetics Education and Development Centre that the collection of new knowledge in an education needs setting did not require NHS ethics approval. The Centre operates in the governance framework of the Birmingham Women's NHS Foundation Trust.

### Validity and reliability

All planning stages were logged in a dedicated research file, informed by a standard project management framework. Authentic stories, selected against agreed criteria, were used to remove any bias that might be associated with constructing scenarios. Assigning individuals to groups assured a mix of backgrounds, promoting a shared understanding of issues. The iteration between groups further strengthened this and ameliorated the risk of any one group dominating the proceedings. The approach, managed by an independent facilitator, ensured that the process was transparent to participants. Finally, participants were invited to confirm that the revised framework was a fair representation of discussions.

## Results

### Participants

Thirty participants (6 male: 24 female) attended the meeting. Thirteen were drawn from clinical practice (including genetics services), 10 from nurse education, four were patient representatives and the remaining three represented the NMC, RCN and NHS National Genetics Education and Development Centre. At the start of the meeting (*n* = 27), 17 (63·0%) had been aware of the genetics education framework prior to the invitation, whilst 10 (37%) had not. When asked about the extent to which nursing care currently was compromised by nurses' level of genetic competence, 23 (82·1%) of those who voted (*n* = 28) felt that care was compromised to some extent and five (17·9%) were unsure.

### Relevance of framework

**Figure 5:**
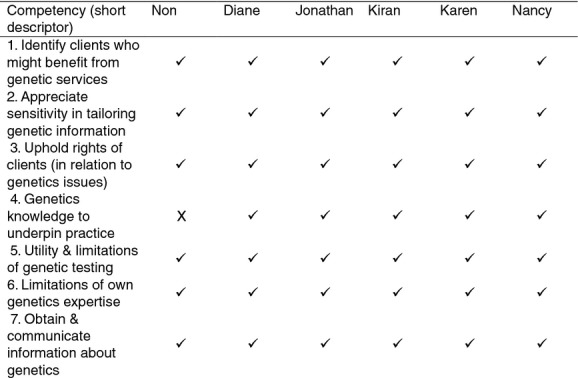
Mapping the stories to the competencies.

Issues relating to the nursing role (What does the nurse need to know, think or do?) were identified for all six stories and are shown along with example verbatim quotes from each worksheet (Table 2). Issues raised mapped to all seven competency statements and all stories except one reflected all seven competencies (Figure 5). Competency statement 4 (Genetics knowledge to underpin practice) was considered implicit rather than explicitly reflected in Non's story. The comprehensive mapping demonstrates that the seven competency statements are relevant to meeting the needs of individuals and families. Groups did not delete any comments on the worksheets.

**Table 2:** Issues and omissions identified by participants in relation to each story, with illustrative quotes

Storyteller	Issues	Omissions to be addressed
Non	Advocacy; ‘OK not to know’; Coordinating role; Awareness of triggers during nursing assessment; Accessing information/confidentiality; Impact of long-term conditions	Competency 2 – importance of language level and cognitive ability
*Advocate for patient autonomy, particularly for vulnerable groups such as children*		
Diane	Advocacy; Ongoing holistic care: coordination of care, needs of the carers; Information: confidentiality, quality; Expert patient and expert carer role; Communication	Competency 3 needs to be more explicit, using the term ‘advocacy’
*Support and understanding of the psychological implications for both parent and child*		
Jonathan	Advocacy; Holistic care; Meeting unrecognized needs; Raising awareness in hard-to-reach groups; Impact & implications of genetic conditions for individual/family; Quality of information; Professional/role boundaries	No additional comments
*Nurse needs the ability to listen effectively and utilize the information from Jonathan – he knows the condition/his family better than anyone else*.		
Kiran	Advocacy; Psychosocial support; Coordination of care; Continuity of care over the life stages; Cultural competence; How to find information	The need for psychological care and support should be emphasized more
*How to give information in a sensitive manner – understanding the possible implications of what has been said - that people have the right not to want to know*		
Karen	Advocacy; Psychosocial support; Coordination of care; Patient as expert; Planning/anticipation of needs over time; Core knowledge for care; Communicating in complex care	Need for more directive terms within competency statements. Coordination of services not covered
*Importance of acknowledgement of needs of all involved in care – needs of carer and of those who are sick. ‘Carers’ as people can be lost and need support*		
Nancy	Importance of triggers in routine assessment; Psychological support around testing; Wider implications of testing; Recognizing limits of genetics expertise	Need for a ‘step before’ Competency 1
*Nurses need to capture the information at assessment that might trigger more in-depth family history assessment or other investigation*		

### Improving the framework: omissions and deficiencies

Although the six stories selected were diverse, common themes were identified when rapporteurs highlighted the key issues in each (Table 2). Thirteen key themes were recorded on the flip chart, linked where possible to the competency statement deemed most relevant. From these, the plenary group developed three overarching themes that they felt were inadequately addressed in the existing framework and merited further discussion: Advocacy, Ongoing care and Information management (Table 3).

**Table 3:** Key themes and overarching categories

Key themes	Competency identified as most relevant	Overarching categories identified for further discussion and voting
Advocacy role: Importance, Duration	3	Advocacy
Psychological care	5	Ongoing care
Service coordination (multidisciplinary team & multiagency)	6	
Patient/family development (trajectory of individual/family journey over time)	6	
Carers' needs (broad definition)	?	
Ongoing management of condition and implications	5	
Respond to patient needs	5	
Patient expertise (recognizing and using)	2	
Quality assurance of (sensible and intelligent) information for staff and patients	7	Information management
Overreliance on IT	7	
Confidentiality	5	None identified
Triggers to make nurses ‘think genetics’	1	
OK not to know, but act	6	

**Figure 6:**
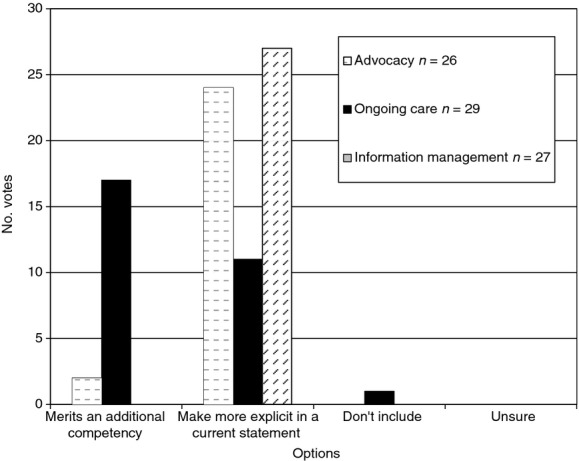
Participants' views on options to address the three overarching themes.

**Figure 7:**
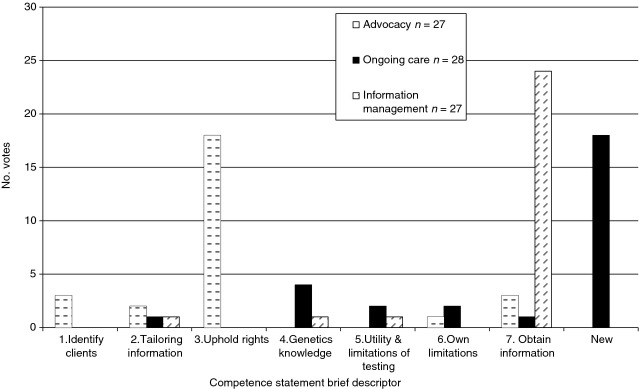
Participants' views on allocation of the three overarching themes to competency statements.

Participants identified different approaches to how the overarching themes should be addressed in a revised framework. All participants were given the opportunity to vote, although there were abstentions. The majority who voted (24/26) felt that Advocacy should be made more explicit in an existing competency, as did all (27/27) regarding Information management (Figure 6). For Advocacy, Competency 3 (Uphold the rights of all clients to informed decision-making) was the majority choice (18/27). Competency 7 (Obtain and communicate credible, current information about genetics) was the majority choice for Information management (Figure 7). Views were mixed about Ongoing care; just over half (17/29) agreeing that it merited an additional statement, 11/29 thinking that it should be made more explicit in a current competency (Figure 6). There was no consensus on which competency it should be included with, 18/28 (64·3%) voting for it to remain separate (Figure 7).

### Potential impact of scientific or policy advances

Participants identified several developments in genomics as potential drivers for implementation of the framework without necessitating their explicit inclusion. The impact of an increase in direct to consumer testing and whole genome scanning were highlighted for potential impact on nurses as individuals seek explanations about test results. The move of genetics/genomics into mainstream provision was also acknowledged to have implications for the nursing role. It was agreed that this should be reflected in the new framework, with specific inclusion of the term ‘genomics’. The dearth of role models in practice was a mutual concern and the importance of continuing professional development for qualified staff was highlighted.

In considering proposed changes to the pre-registration requirements (Nursing & Midwifery Council 2010), opinions were divided as to whether both draft statements related to genetics were sufficient to inform the development of appropriate curriculum content to achieve the competencies discussed. Just over half (14/25) felt they were not sufficient, 11/25 felt that they were. None agreed the statements provided more information than necessary. In commenting on the voting outcome, one participant clarified that the statements would be sufficient for those with ‘prior insight’ into genetics/genomics, but that this would not apply to the majority.

### The revised framework

Participants were sent the key revisions identified from the field notes, worksheets and voting responses (Table 4), along with the proposed framework. All statements were revised. In particular, Competency 1 now reflects the need to include family history information as a part of a comprehensive nursing assessment and Competency 6 emphasizes the responsibility of nurses to keep updated in their practice. An additional statement (Competency 8) highlights the importance of ongoing nursing care to address the needs of individuals and their family/carers that may change over time. Twenty-six participants (86·6%) responded and all approved the changes. Responses were positive:

**Table 4:** Key changes to the original competency statements

Competency	Key changes required
1	‘Identifying patients’ should be highlighted as something nurses should do as part of a systematic and comprehensive assessment, with nurses being aware of the ‘red flags’ that should alert them to possible further action
2	Language level and developmental stage are important issues
3	Reflect a more proactive approach, with nurses being prepared to advocate on behalf of patients (acknowledging that this term has a slightly different meaning in learning disability nursing)
4	No major change: the importance of core knowledge & skills to underpin practice was endorsed; the term ‘genomics’ should be included
5	Reflect the need for a more proactive approach (apply to practice, rather than demonstrate knowledge of…) to the whole process around genetic testing. Psychological and social consequences needed to be emphasized more by referring to them individually
6	Reflect the need for nurses not only to recognize their limitations, but also to address identified gaps through continued professional development
7	Make clearer the need to identify other sources of information and to apply critical appraisal skills in making judgements on the quality of information
New statement	Importance of the nursing role in providing ongoing care, inadequately captured in the existing framework. Components identified are of sufficient weight to merit an additional competency statement

I do feel the competence framework is exactly what we discussed on the day. [Clinician]

Well done on synthesizing our varied thoughts into a clear and simple statement of intent. [Clinician]

The final approved framework is shown (Table 5).

**Table 5:** Nursing competencies in genetics/genomics: revised framework

1	Identify individuals who might benefit from genetic services and/or information through a comprehensive nursing assessment: that recognizes the importance of family history in assessing predisposition to disease, recognizing the key indicators of a potential genetic condition, taking appropriate and timely action to seek assistance from, and refer individuals to, genetics specialists, other specialists and peer support resources, based on an understanding of the care pathways that incorporate genetics services and information.
2	Demonstrate the importance of sensitivity in tailoring genetic/genomic information and services to the individual's culture, knowledge, language ability and developmental stage: recognizing that ethnicity, culture, religion, ethical perspectives and developmental stage may influence the individual's ability to use information and services, demonstrating the use of appropriate communication skills in relation to the individual's level of understanding of genetic/genomic issues.
3	Advocate for the rights of all individuals to informed decision-making and voluntary action: based on an awareness of the potential for misuse of human genetic/genomic information, understanding the importance of delivering genetic/genomic education and counselling fairly, accurately and without coercion or personal bias, recognizing that personal values and beliefs of self and individuals may influence the care and support provided during decision-making and that choices and actions may differ over time.
4	Demonstrate a knowledge and understanding of the role of genetic/genomic and other factors in maintaining health and in the manifestation, modification and prevention of disease expression, to underpin effective practice: which includes core genetic/genomic concepts that form a sufficient knowledge base for understanding the implications of specific conditions that may be encountered.
5	Apply knowledge and understanding of the utility and limitations of genetic/genomic information and testing to underpin care and support for individuals and families prior to, during and following decision-making, that: incorporates awareness of the ethical, legal and social issues related to testing, recording, sharing and storage of genetic/genomic information, incorporates awareness of the potential physical, emotional, psychological and social consequences of genetic/genomic information for individuals, family members and communities.
6	Examine one's own competency of practice on a regular basis: recognizing areas where professional development related to genetics/genomics would be beneficial, maintaining awareness of clinical developments in genetics/genomics that are likely to be of most relevance to the client group, seeking further information on a case-by-case basis, based on an understanding of the boundaries of one's professional role in the referral, provision or follow-up to genetics services.
7	Obtain and communicate credible, current information about genetics/genomics, for self, patients, families and colleagues: using information technologies and other information sources effectively to do so and applying critical appraisal skills to assess the quality of information accessed.
8	Provide ongoing nursing care and support to patients, carers and families with genetic/genomic healthcare needs: being responsive to changing needs through the life stages and during periods of uncertainty, demonstrating awareness about how an inherited condition and its implications for family members might have an impact on family dynamics, working in partnership with family members and other agencies in the management of conditions, recognizing the potential expertise of individuals, family members and carers with genetic/genomic healthcare needs that develops over time and with experience.

### Learning outcomes

Using an approach taken previously (Skirton *et al*. 2006), learning outcomes and practice indicators were written for all eight statements in a format allowing integration into undergraduate nursing programmes (see Table S2). Outcomes are linked to Levels 4, 5 and 6 of the QAA Framework (The Quality Assurance Agency for Higher Education 2008), corresponding to years 1, 2 and 3, respectively, of a pre-registration undergraduate nursing programme. Outcomes build on those of the previous year and the indicators for practice alongside the learning outcomes allow a means of measuring and confirming that competence has been attained.

The group acknowledged that nursing curricula are under pressure to include other content. They identified topic areas traditionally present in nursing curricula where genetics/genomics content could be incorporated. This additional guidance, along with signposting to appropriate resources, is being developed.

## Discussion

Expert consensus is a valuable approach when research evidence to inform guidelines is limited (Raine *et al*. 2005). A facilitated participative approach is important for a complex field such as genomic health care, allowing exchange of views and clarification. With a face-to-face meeting, iteration and expert facilitation in a structured programme, the approach we used shares many essential elements of the nominal group technique (Delbecq & van de Ven 1971). However, the structured group work and use of stories as cues to address specific questions are a deviation from the standard technique. In their systematic review, Murphy *et al*. (1998) highlight a potential problem of including scenarios, as they could be perceived as unrealistic or irrelevant. Real-life, verbatim narratives overcome this. Notably, all groups became immersed in the stories and discussion continued through programmed breaks. Groups made detailed notes on the worksheets, responding to previous comments using ticks, or linking comments with arrows, lines and asterisks. Exit and subsequent feedback indicated that participants had found the event to be interesting and thought-provoking and the authors recommend this approach in promoting engagement.

Themes were generated by participant thematic analysis from the stories and only subsequently compared against the original competency framework to assess ‘fit’. This was an important element, making transparent any themes that the framework did not adequately address. This also demonstrated the relevance of the competencies to patient needs and helped to conserve the original framework where appropriate; therefore, not appearing wholly unfamiliar to existing users.

The review indicated that the competency statements were a valid reflection of patient needs and of the nursing role in meeting them. They were incomplete, however, particularly in capturing the nursing role in providing ongoing care. The inclusion of the eighth competency is perhaps indicative of the growing awareness of the responsibilities of nurses in the ‘clinical mainstream’ to integrate genetics/genomics throughout an episode of care. Whilst the need to ‘Identify individuals who might benefit from genetic services…’ (Competency 1) is undoubtedly a crucial first step in specialist genetics referral, the responsibilities of the nurse do not end there. This is an important addition to the competencies, as endorsed by one participant:

As a patient, number 8 is a very welcome addition and covers it pretty perfectly.

The revised framework reflects a more holistic approach to care and acknowledges the role of individuals and families as (often expert) partners in care. However, a crucial element in promoting engagement with the revised framework is the availability of relevant, accessible resources to which educators and learners can be directed (Tonkin *et al*. 2011). Further work is needed to develop this.

### Implications for nursing education and practice

It is interesting that participants felt that the terms used in the revised framework should be more directive. This perhaps reflects progress towards nurses embracing genetics/genomics since the first framework in 2003. Then, the emphasis was on encouraging nurses to become more aware of genetics and to view their practice ‘through the genetics lens’ (Kirk *et al*. 2003, p50). In the intervening years, advances in genomics and its gradual move into the healthcare mainstream have sharpened minds, reflected in the forthright argument that ‘There is no dispute that nurses must understand basic concepts of genetics and genomics. Genetics and genomics are a necessary part of nursing curricula’ (Giarelli & Reiff 2012, p529). There is an ongoing need for competency guidelines. A limitation of the Nursing and Midwifery Council (2010) requirements is a lack of guidance on managing the findings of a nursing assessment that considers genetic factors, both in terms of planning subsequent care and the ethical framework where such an assessment and the findings should be managed. It is worth noting that, in its final report, the NMC removed the reference to genetics as essential content to underpin practice (included in the voting by the participants in this study).

This work is of international relevance. While many studies have measured nurses' perceived knowledge and confidence, few measure aspects of competence (Skirton *et al*. 2012). However, even where genetic health care has been established clinically for decades, there is evidence that nurses lack sufficient awareness of basic genetic concepts to offer appropriate care to patients across a range of specialties, including primary care. For example, recent work by Godino *et al*. (2013) in Italy indicated that while the majority of nurses were able to answer simple questions about genetic influences on disease, only a minority believed that genetics was relevant to their role. Even where nurses are taught genetics, this may not be applied to practice due to the teacher's limited knowledge or experience (Jenkins & Calzone 2012), leading to an inability to apply theoretical topics to the clinical setting. Work is therefore needed in many countries not only to establish relevant competencies, but to apply these to real-life clinical situations and ensure that educators can prepare nurses in the theory and practice of genetic and genomic health care. Further research into the levels of competence of nurses [such as the studies conducted by Bottorff *et al*. (2005) and Godino *et al*. (2013)] would provide a stronger evidence base to support the call for genetics/genomics to be better integrated into the nursing curriculum.

### Limitations

One of the limitations of any consensus approach using a selected group of participants is its reliability, with the potential for discussion and decisions being unrepresentative (Raine *et al*. 2005). The selection of participants, the rigour of the interactive approach and involvement of an expert facilitator sought to alleviate potential limitations. Potential for bias in the selection of stories was addressed through applying a matrix of criteria seeking to optimize the representativeness of the stories in capturing a diversity of patient needs. More stories could have been used had more time been available and this was a pragmatic decision.

## Conclusion

The consensus method that we adopted promoted engagement in a complex issue across a diverse group and provided a constructive means to demonstrate both the relevance and deficiencies of the competency framework. The revised framework is more contemporary and comprehensive, with more detailed learning outcomes, relevant to the nursing role and informed by individual/family needs. Both the approach and the framework may be of value to other areas of nursing in the UK and internationally. In the latter case, Skirton *et al*. (2010) advise that competency frameworks must be adapted to the national and cultural context where nurses are practising. The critical success factor in engaging with the genetics/genomics competency framework, however, is active nurse leadership, demonstrated by changes to policy, education and practice.

## Supporting Information Online

Table S1 Nursing competencies in genetics (Kirk et al. 2003).

Table S2 Learning outcomes and practice indicators for each nursing competency in genetics/genomics.
