# Supplementary material for: An iterative consensus-building approach to revising a genetics/genomics competency framework for nurse education in the UK
Source: J Adv Nurs. 2013 Jul 23;70(2):405–20. doi: 10.1111/jan.12207 (PMC3907026; doi:10.1111/jan.12207)
Supplement: Table S1 — Nursing competencies in genetics (Kirk et al. 2003). [file jan0070-0405-sd1.docx]

**Supplemental material Table 1** Nursing competencies in genetics (Kirk *et al.* 2003)

All nurses, midwives and health visitors, at the point of registration, should be able to:

1. **Identify clients who might benefit from genetic services and information**

- through an understanding of the importance of family history in assessing predisposition to disease,
- seeking assistance from and referring to appropriate genetics experts and peer support resources, and
- based on an understanding of the components of the current genetic counselling process.

1. **Appreciate the importance of sensitivity in tailoring genetic information and services to clients’ culture, knowledge and language level**

- recognising that ethnicity, culture, religion and ethical perspectives may influence the clients' ability to utilise these.

1. **Uphold the rights of all clients to informed decision making and voluntary action**

- based on an awareness of the history of misuse of human genetic information and
- understanding of the importance of delivering genetic education and counselling fairly, accurately and without coercion or personal bias,
- recognising that personal values and beliefs may influence the care and support provided to clients during decision-making.

1. **Demonstrate a knowledge and understanding of the role of genetic and other factors in maintaining health and in the manifestation, modification and prevention of disease expression, to underpin effective practice.**
2. **Demonstrate a knowledge and understanding of the utility and limitations of genetic testing and information**

- including the ethical, legal and social issues related to testing and recording of genetic information and
- the potential physical and/or psychosocial consequences of genetic information for individuals, family members, and communities.

1. **Recognise the limitations of one’s own genetics expertise**

- based on an understanding of one’s professional role in the referral, provision or follow-up to genetics services.

1. **Obtain and communicate credible, current information about genetics, for self, clients and colleagues**
   - using information technologies effectively to do so.
