# Supplementary material for: An iterative consensus-building approach to revising a genetics/genomics competency framework for nurse education in the UK
Source: J Adv Nurs. 2013 Jul 23;70(2):405–20. doi: 10.1111/jan.12207 (PMC3907026; doi:10.1111/jan.12207)
Supplement: Table S2 — Learning outcomes and practice indicators for each nursing competency in genetics/genomics. [file jan0070-0405-sd2.docx]

**Supplemental material Table 2** Learning outcomes and practice indicators for each nursing competency in genetics/genomics

| **1. Identify individuals who might benefit from genetic services and/or information through a comprehensive nursing assessment:**   - that recognises the importance of family history in assessing predisposition to disease, - recognising the key indicators of a potential genetic condition, - taking appropriate and timely action to seek assistance from and refer individuals to genetics specialists, other specialists and peer support resources,   based on an understanding of the care pathways that incorporate genetics services and information. | | | |
| --- | --- | --- | --- |
| **Level 4 Learning Outcomes (Year 1)** | **Level 5 Learning Outcomes (Year 2)** | **Level 6 Learning Outcomes (Year 3)** | **Practice Indicators** |
| 1.1.1 Outline the relevance of a multi-generational family history in relation to assessing genetic health risk.  1.1.3 Describe basic patterns of biological inheritance and their variation in families and populations.  1.1.4 List resources available for clients and professionals seeking genetic information.  1.1.5 Identify the different types of services and other agencies which can support individuals and families with or at risk of a genetic condition | 1.2.1 Acquire and record accurate information for the construction of a multi-generational family history to assess genetic health risk.  1.2.2 Explore significant family history to recognise genetic risk of altered health states.  1.2.3 Distinguish patterns of biological inheritance and their variation in families and populations.  1.2.4 Utilise relevant genetic information resources to inform practice.  1.2.5 Describe the roles of specialist genetic practitioners and the range of services they provide | 1.3.1 Construct a multi-generational family history for the process of assessing genetic health risk.  1.3.2 Interpret significant family history to assess genetic risk of altered health states.  1.3.3 Differentiate patterns of biological inheritance & explain how these may vary in families and populations.  1.3.4 Use and evaluate relevant genetic information resources to inform practice.  1.3.5 Appraise the role of specialist genetic services and other agencies in the provision of appropriate patient/client care.  1.3.6 Describe a typical patient pathway which incorporates genetic services and information | Demonstrate the ability to collect information as part of a comprehensive nursing assessment and use the information to draw a family history using standard symbols.  Recognise and document potentially significant genetic/genomic information from a family history.  Apply knowledge of local and regional referral pathways to explain to patients the services that are available.  Facilitate referral to genetic services and other agencies when appropriate. |

| **2. Demonstrate the importance of sensitivity in tailoring genetic/genomic information and services to the individual’s culture, knowledge, language ability and developmental stage:**   - recognising that ethnicity, culture, religion, ethical perspectives and developmental stage may influence the individual’s ability to utilise information and services, - demonstrating the use of appropriate communication skills in relation to the individual’s level of understanding of genetic/genomic issues. | | | |
| --- | --- | --- | --- |
| **Level 4 Learning Outcomes (Year 1)** | **Level 5 Learning Outcomes (Year 2)** | **Level 6 Learning Outcomes (Year 3)** | **Practice Indicators** |
| 2.1.1 Recognise how communication of genetics issues should take into account the client’s level of understanding.  2.1.2 Outline how a client’s ethnicity, culture, religion and ethical perspectives may influence their understanding and use of genetic information and services. | 2.2.1 Demonstrate the use of appropriate communication skills in relation to the client’s level of understanding of genetics issues.  2.2.2 Discuss the impact of ethnicity, culture, religion and, ethical perspectives on a client’s potential use of genetic information and services. | 2.3.1 Effectively communicate genetic issues at a client’s level of understanding.  2.3.2 Critically evaluate the significance of ethnicity, culture, religion and ethical perspectives on a client’s potential use of genetic information and services. | Demonstrate the ability to communicate sensitively with clients to elucidate their ethnic, cultural, religious and ethical perspectives.  Demonstrate sensitive and effective communication of genetic issues.  Tailor genetic information to meet individual needs taking into account the cultural, ethnic, religious and ethical perspectives as well as developmental stage, and using resources to facilitate effective communication as appropriate.  Identify and assess an individual’s understanding of genetic information.  Describe how the impact of information is influenced by individual factors. |

| **3. Advocate for the rights of all individuals to informed decision making and voluntary action:**   - based on an awareness of the potential for misuse of human genetic/genomic information, - understanding the importance of delivering genetic/genomic education and counselling fairly, accurately and without coercion or personal bias, - recognising that personal values and beliefs of self and individuals may influence the care and support provided during decision-making, and that choices and actions may differ over time. | | | |
| --- | --- | --- | --- |
| **Level 4 Learning Outcomes (Year 1)** | **Level 5 Learning Outcomes (Year 2)** | **Level 6 Learning Outcomes (Year 3)** | **Practice Indicators** |
| 3.1.1 Explain how one’s own beliefs and values can influence client care.  3.1.2 Identify past and potential future misuse of genetic information.  3.1.3 Recognise the rights of all individuals to informed decision making and voluntary action. | 3.2.1 Explore how personal values and beliefs in relation to ethical, cultural, religious and ethnic issues could impact on client care.  3.2.2 Discuss how the misuse of genetic information could potentially influence a client’s ability to make an informed decision and act voluntarily.  3.2.3 Facilitate clients’ rights to self determination through ensuring informed decision making and voluntary action.  3.2.4 Ensure that the particular needs of those unable to give informed consent in relation to accessing genetic information are addressed. | 3.3.1 Critically evaluate the significance of personal values and beliefs in relation to ethical, cultural, religious and ethnic issues in the context of client care.  3.3.2 Appraise the impact of genetic information misuse on a client’s ability to make an informed decision and take voluntary action.  3.3.3 Uphold clients’ rights to self determination through ensuring informed decision making and voluntary action.  3.3.4 Advocate the particular needs of those unable to give informed consent in relation to accessing genetic information.  3.3.5 Analyse how the principle of a non-directive approach underpins the process of genetic counselling, in facilitating client autonomy and empowerment. | Articulate situations where people’s values and beliefs might impact on care.  Identify situations where patients/clients may be vulnerable to coercion and involuntary action.  Assess patient’s ability to make informed decisions and triggers best interest process.  Where or when appropriate, act as an advocate or work with advocacy agencies. |

| **4. Demonstrate a knowledge and understanding of the role of genetic/genomic and other factors in maintaining health and in the manifestation, modification and prevention of disease expression, to underpin effective practice:**   - which includes core genetic/genomic concepts that form a sufficient knowledge base for understanding the implications of specific conditions that may be encountered. | | | |
| --- | --- | --- | --- |
| **Level 4 Learning Outcomes (Year 1)** | **Level 5 Learning Outcomes (Year 2)** | **Level 6 Learning Outcomes (Year 3)** | **Practice Indicators** |
| 4.1.1 Discuss how genetic and genomic factors affect health and disease.  4.1.2 Outline how disease expression throughout the life cycle is affected by both genetic and genomic factors. | 4.2.1 Explore the role of genetic and genomic factors in altered health states using examples of common inherited conditions.  4.2.2 Explain with examples, how disease expression throughout the lifecycle may be influenced by genetic and genomic factors. | 4.3.1 Apply knowledge of genetic and genomic factors within the human health-disease continuum, including in the context of public health.  4.3.2 Critically analyse the impact of genotype and environment throughout the human life cycle.  4.3.4 Distinguish between genetic susceptibility and clinical manifestation of disease using basic concepts of risk. | Explain the genetic component contributing to the manifestation of disorders within their sphere of practice.  Provide appropriate lifestyle advice based on knowledge of gene-environment interactions.  Demonstrate ability to distinguish between individuals at high, medium and low risk of complex conditions. |

| **5. Apply knowledge and understanding of the utility and limitations of genetic/genomic information and testing to underpin care and support for individuals and families prior to, during and following decision-making, that:**   - incorporates awareness of the ethical, legal and social issues related to testing, recording, sharing and storage of genetic/genomic information, - incorporates awareness of the potential physical, emotional, psychological and social consequences of genetic/genomic information for individuals, family members, and communities. | | | |
| --- | --- | --- | --- |
| **Level 4 Learning Outcomes (Year 1)** | **Level 5 Learning Outcomes (Year 2)** | **Level 6 Learning Outcomes (Year 3)** | **Practice Indicators** |
| 5.1.1 Explain the need for and maintain privacy and confidentiality when discussing and recording genetic information.  5.1.2 Demonstrate awareness of the process of genetic testing and its limitations.  5.1.3 Demonstrate awareness that individuals and families may have ongoing needs for support in relation to the genetic condition. | 5.2.1 Demonstrate confidentiality and maintain privacy when discussing and recording genetic information.  5.2.2 Explore potential risks, benefits and limitations of genetic testing and access to genetic information.  5.2.3 Debate the psychological, ethical, legal and social implications of genetic information for individuals and families.  5.2.4 Respond appropriately to enquiries about genetic concerns. | 5.3.1 Ensure confidentiality and privacy when discussing and recording genetic information.  5.3.2 Evaluate potential risks, benefits and limitations of genetic testing and access to genetic information.  5.3.3 Critically appraise the psychological, ethical, legal and social implications of genetic information for individuals and families.  5.3.4 Respond appropriately and effectively to enquiries about genetic concerns recognising the limitations of one’s own knowledge.  5.3.5 Recognises individuals and families who have needs for ongoing support in relation to the genetic condition. | Ensure genetic information is discussed in an appropriate environment.  Obtain consent to share information with other professionals and with other family members as appropriate.  Demonstrate awareness of the potential psychological effects of accepting or declining genetic testing on the individual and family.  Evaluate the appropriateness of genetic information for individuals.  Communicate information in an appropriate and sensitive way, involving appropriate health professional(s) as necessary.  Where appropriate, provides ongoing support to individuals and families. |

| **6. Examine one’s own competency of practice on a regular basis:**   - recognising areas where professional development related to genetics/genomics would be beneficial, - maintaining awareness of clinical developments in genetics/genomics that are likely to be of most relevance to the client group, seeking further information on a case-by-case basis, - based on an understanding of the boundaries of one’s professional role in the referral, provision or follow-up to genetics services. | | | |
| --- | --- | --- | --- |
| **Level 4 Learning Outcomes (Year 1)** | **Level 5 Learning Outcomes (Year 2)** | **Level 6 Learning Outcomes (Year 3)** | **Practice Indicators** |
| 6.1.1 Recognise the opportunities for learning related to genetics/genomics knowledge and practice.  6.1.2 Recognise the limitations of your role in the referral, provision or follow-up to genetic services. | 6.2.1 Utilise learning resources to improve genetic/genomic knowledge and practice.  6.2.2 Develop a collaborative approach to patient/client care in relation to genetics, within a multidisciplinary team including other statutory and voluntary organisations. | 6.3.1 Maintain contemporaneous knowledge of genetic/genomic developments and the implications for your practice.  6.3.2 Promote a collaborative approach to enhance patient/client care in relation to genetics with other statutory and voluntary organisations. | Demonstrate ongoing professional development in genetics/genomics within portfolio.  Demonstrate an awareness of the boundaries of self and others involved in the provision of genetic care.  Enhance patient care through working collaboratively with other service providers. |

| **7. Obtain and communicate credible, current information about genetics/genomics, for self, patients, families and colleagues:**   - using information technologies and other information sources effectively to do so, and - applying critical appraisal skills to assess the quality of information accessed. | | | |
| --- | --- | --- | --- |
| **Level 4 Learning Outcomes (Year 1)** | **Level 5 Learning Outcomes (Year 2)** | **Level 6 Learning Outcomes (Year 3)** | **Practice Indicators** |
| 7.1.1 Employ a range of appropriate genetic/genomic information resources to inform practice.  7.1.2 Recognise the importance of regularly updating genetics knowledge from reputable sources.  7.1.3 Display an ability to use information technology to retrieve relevant and reliable genetic/genomic information. | 7.2.1 Evaluate a range of appropriate genetic/genomic information resources to inform practice.  7.2.2 Evaluate and incorporate current genetic knowledge from reputable sources into practice.  7.2.3 Utilise reliable genetic/genomic evidence when communicating with patient/clients. | 7.3.1 Critically evaluate information and evidence from a range of reliable sources  7.3.2 Critically evaluate and incorporate current reputable genetic information into own practice.  7.3.3 Develop effective communication strategies to inform clients and colleagues of relevant genetic/genomic information. | Demonstrate ability to select reliable and appropriate genetic/genomic information.  Demonstrate ability to utilise reliable and appropriate genetic/genomic information.  Demonstrate effective communication skills when discussing genetic/genomic information with clients and colleagues. |

| **8. Provide ongoing nursing care and support to patients, carers and families with genetic/genomic healthcare needs:**   - being responsive to changing needs through the life-stages and during periods of uncertainty, - demonstrating awareness about how an inherited condition, and its implications for family members, might impact on family dynamics, - working in partnership with family members and other agencies in the management of conditions, - recognising the potential expertise of individuals, family members and carers with genetic/genomic healthcare needs that develops over time and with experience. | | | |
| --- | --- | --- | --- |
| **Level 4 Learning Outcomes (Year 1)** | **Level 5 Learning Outcomes (Year 2)** | **Level 6 Learning Outcomes (Year 3)** | **Practice Indicators** |
| 8.1.1 Describe the key life stages where a genetic diagnosis or condition may have an impact.  8.1.2 Describe basic patterns of biological inheritance and their variation in families. (1.1.3)  8.1.3 Describe the roles of key members of multi-agency teams involved in the care of people with enduring genetic healthcare needs.  8.1.4 Recognise that an individual and/or family member may have expertise about a particular genetic condition. | 8.2.1 Explain how a genetic condition may impact at different life stages within a family.  8.2.2 Apply knowledge of inheritance patterns to identify members within a family who might have or be at risk of a genetic condition.  8.2.3 Discuss how a multi-agency team might interact in providing ongoing care.  8.2.4 Discuss how expertise within the family can inform ongoing care. | 8.3.1 Apply knowledge and understanding of impact of genetic conditions at different life stages to plan care and anticipate family needs.  8.3.2 Integrate understanding of inheritance risk with knowledge of potential bio-psychosocial consequences of genetic/genomic information, to outline potential impact on family dynamics.  8.3.3 Promote effective interaction within a multidisciplinary team to coordinate care.  8.3.4 Promote a partnership approach with the individual and/or family members as appropriate to ensure optimal care  8.3.5 Utilise the expertise of the individual and/or family members to gain knowledge and understanding of a particular genetic condition for self and others. | Demonstrate knowledge and understanding of genetics and genomics appropriate to life stages, when carrying out a nursing assessment and care delivery.  Incorporate knowledge of inheritance risk and impact on family dynamics to anticipate potential issues when planning care.  Recognise own role and contribute effectively within a team. Contribute to the co-ordination of an individual’s care, demonstrating leadership as appropriate.  Actively seek advice from the individual or family members to address care needs.  Demonstrate ability to work in partnership with families. |
